# Correction: The role of electrochemical biosensors in SARS-CoV-2 detection: a bibliometrics-based analysis and review

**DOI:** 10.1039/d2ra90107b

**Published:** 2022-11-02

**Authors:** Shuduan Mao, Li Fu, Chengliang Yin, Xiaozhu Liu, Hassan Karimi-Maleh

**Affiliations:** Key Laboratory of Pollution Exposure and Health Intervention of Zhejiang Province, Interdisciplinary Research Academy (IRA), Zhejiang Shuren University Hangzhou 310015 China; Key Laboratory of Novel Materials for Sensor of Zhejiang Province, College of Materials and Environmental Engineering, Hangzhou Dianzi University Hangzhou 310018 China fuli@hdu.edu.cn; National Engineering Laboratory for Medical Big Data Application Technology, Chinese PLA General Hospital Beijing China; Medical Big Data Research Center, Medical Innovation Research Division of PLA General Hospital Beijing China; Department of Cardiology, The Second Affiliated Hospital of Chongqing Medical University Chongqing 400010 China; School of Resources and Environment, University of Electronic Science and Technology of China Xiyuan Ave 611731 Chengdu China; Department of Chemical Engineering, Quchan University of Technology Quchan 9477177870 Iran; Department of Chemical Sciences, University of Johannesburg Doornfontein Campus, 2028 Johannesburg 17011 South Africa

## Abstract

Correction for ‘The role of electrochemical biosensors in SARS-CoV-2 detection: a bibliometrics-based analysis and review’ by Shuduan Mao *et al.*, *RSC Adv.*, 2022, **12**, 22592–22607, https://doi.org/10.1039/D2RA04162F.

The authors regret that the name and affiliation of the first author (Shuduan Mao) was shown incorrectly in the original article. The corrected author list and affiliations are as shown here.

The Royal Society of Chemistry apologises for these errors and any consequent inconvenience to authors and readers.

## Supplementary Material

